# Association of antibiotic exposure with residual cancer burden in HER2-negative early stage breast cancer

**DOI:** 10.1038/s41523-024-00630-w

**Published:** 2024-03-26

**Authors:** Amit A. Kulkarni, Aditya Jain, Patricia I. Jewett, Nidhi Desai, Laura Van ’t Veer, Gillian Hirst, Douglas Yee, Anne H. Blaes, ISPY2 consortium

**Affiliations:** 1 Masonic Cancer Center, University of Minnesota, Minneapolis, MN USA; 2 Division of Hematology, Oncology, and Transplantation, University of Minnesota, Minneapolis, MN USA; 3 Department of Medicine, University of Minnesota, Minneapolis, MN USA; 4 Helen Diller Family Comprehensive Cancer Center, University of California, San Francisco, CA USA

**Keywords:** Breast cancer, Cancer immunotherapy

## Abstract

Antibiotic exposure during immunotherapy (IO) has been shown to negatively affect clinical outcomes in various cancer types. The aim of this study was to evaluate whether antibiotic exposure in patients with high-risk early-stage HER2-negative breast cancer (BC) undergoing treatment with neoadjuvant pembrolizumab impacted residual cancer burden (RCB) and pathologic complete response (pCR) in the pembrolizumab-4 arm of the ISPY-2 clinical trial. Patients received pembrolizumab for four cycles concurrently with weekly paclitaxel for 12 weeks, followed by four cycles of doxorubicin plus cyclophosphamide every 2 or 3 weeks. Patients who received at least one dose of systemic antibiotics concurrently at the time of immunotherapy (IO) were included in the antibiotic exposure group (ATB+). All other participants were included in the control group (ATB-). RCB index and PCR rates were compared between the ATB+ and ATB- groups using *t*-tests and Chi-squared tests, and linear and logistic regression models, respectively. Sixty-six patients were included in the analysis. 18/66 (27%) patients were in the ATB+ group. Antibiotic use during IO was associated with a higher mean RCB index (1.80 ± 1.43 versus 1.08 ± 1.41) and a lower pCR rate (27.8% versus 52.1%). The association between antibiotic use and the RCB index remained significant in multivariable linear regression analysis (RCB index-coefficient 0.86, 95% CI 0.20–1.53, *P* = 0.01). Our findings suggest that concurrent antibiotic exposure during neoadjuvant pembrolizumab in HER2-negative early-stage BC is associated with higher RCB. Further validation in larger cohorts is needed to confirm these findings.

## Introduction

The gastrointestinal (gut) microbiome plays a key role in the immune system^1,2^. Antibiotics can cause disruptions in gut composition and immune dysregulation and therefore may have the potential to impact responses to cancer immunotherapy (IO)^3^. Antibiotic exposure during treatment with immune checkpoint inhibitors (ICI) has been associated with inferior outcomes including decreased progression-free survival (PFS) and overall survival (OS) in metastatic non-small cell lung cancer (NSCLC), renal cell cancer (RCC), urothelial cancer, and melanoma^1,4–7^. Understanding the interplay between antibiotics and the host immune system may be essential to developing more effective immunotherapy and improving clinical outcomes^1^.

Pembrolizumab is an ICI approved for neoadjuvant treatment of high-risk, early-stage triple-negative breast cancer (TNBC)^8^. In the I-SPY2 trial, pembrolizumab use more than doubled the estimated pathologic complete response (pCR) rates for both hormone receptor (HR)-positive/ERBB2-negative and TNBC when added to standard neoadjuvant chemotherapy^9^.

Whether antibiotic use with ICI affects residual cancer burden (RCB) and pCR outcomes during neoadjuvant treatment in early-stage breast cancer (BC) is not known. Therefore, we conducted a secondary analysis within the pembrolizumab-4 arm of the ISPY-2 trial to explore whether antibiotic exposure during neoadjuvant pembrolizumab impacted RCB and pCR.

## Results

### Patient population

Of the 66 participants eligible for this analysis (69 enrolled, one with unknown antibiotic use, and 2 patients with stage I disease were excluded), 18 (27%) patients were in the ATB+ group, with a mean age of 48.3 ± 10.8 years (Table 1). Approximately 70% of all participants had stage II cancer, and approximately 59% of participants were HR-positive (Table 1). Approximately 78.8% of all participants received all four doses of pembrolizumab, with any delays in receiving pembrolizumab among 12.1% of all participants (Table 1). The most frequently used antibiotic classes were fluoroquinolones (ciprofloxacin and levofloxacin; *N* = 16 patients) and cephalosporins (cefazolin, ceftriaxone, and cephalexin; *N* = 16 patients) (Supplementary Table 1).

**Table 1:** Characteristics of the study population, Pembrolizumab-4 arm of the I-SPY2 trial, *N* = 66, 2015–2017

Characteristic	Total (*N* = 66)	No antibiotics during IO (*N* = 48)	Antibiotics during IO (*N* = 18)	
	Mean (std)	Mean (std)	Mean (std)	*P*
**Age at trial enrollment, years**	48.3 (10.8)	48.8 (10.3)	46.8 (12.2)	0.49
	***N*** **(%)**	***N*** **(%)**	***N*** **(%)**	***P***
**Cancer stage**				0.34
II	44 (69.8)	33 (73.3)	11 (61.1)	
III	19 (30.2)	12 (26.7)	7 (38.9)	
missing	3	3	0	
**HR status**				0.28
HR-	28 (41.2)	17 (35.4)	9 (50.0)	
HR+	40 (58.8)	31 (64.6)	9 (50.0)	
**Number of pembrolizumab doses received**				0.53
1	3 (4.6)	1 (2.1)	2 (11.1)	
2	4 (6.1)	3 (6.3)	1 (5.6)	
3	6 (9.1)	4 (8.3)	2 (11.1)	
4	52 (78.8)	39 (81.3)	13 (72.2)	
5	1 (1.5)	1 (2.1)		
**Number of delayed pembrolizumab cycles**				0.75
0	58 (87.9)	41 (85.4)	17 (94.4)	
1	7 (10.6)	6 (12.5)	1 (5.6)	
2	1 (1.5)	1 (2.1)		

### Efficacy analysis

**Fig. 1:**
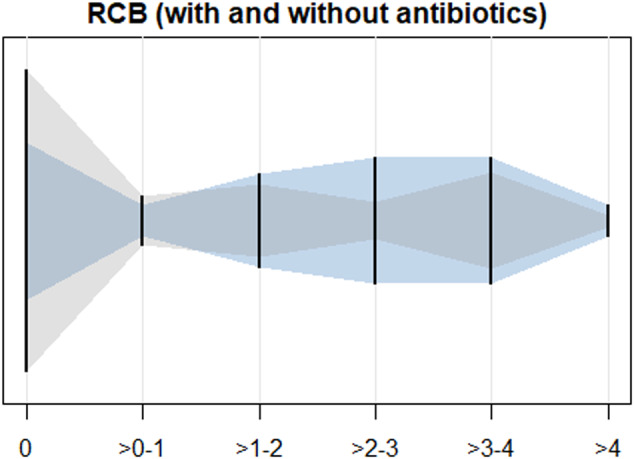
Distribution of Residual Cancer Burden in patient population based on exposure to antibiotics. Distribution of residual cancer burden (RCB) index among those who did (blue) versus did not (gray) receive antibiotics during treatment with Pembrolizumab, Pembrolizumab-4 arm of the I-SPY2 trial, *N* = 66, 2015–2017.

Antibiotic use during IO was associated with a higher mean RCB index (1.80 ± 1.43 versus 1.08 ± 1.41, Fig. 1) and lower pCR rate (38.5% versus 55.6%). The association between antibiotic use and RCB index was significant in multivariable linear regression analysis (RCB index-coefficient 0.86, 95% CI 0.20–1.53, Table 2). The association of antibiotic use with not achieving pCR did not reach significance (Odds ratio 2.83, 95% CI 0.87–9.17). Given limited degrees of freedom, we did not run an adjusted logistic regression model for pCR as the outcome. With ever-antibiotics use as the main exposure, results trended in the same direction as the main model, however without reaching statistical significance (Supplementary Table 2). In a supplemental unadjusted analysis stratified by HR status, differences were significant among those who were HR-positive, but not among those who were HR-negative (Supplementary table 3).

**Table 2:** RCB class distribution by antibiotic use and associations of antibiotics use during immunotherapy with RCB index and pCR, unadjusted and adjusted, Pembrolizumab-4 arm of the I-SPY2 trial, *N* = 66, 2015–2017

RCB class distribution by antibiotics use						
Antibiotics use during IO		RCB class 0	RCB class I	RCB class II	RCB class III	*P*^b^
		*N* (%)	*N* (%)	*N* (%)	*N* (%)	
No		24 (52.2)	8 (17.4)	8 (17.4)	6 (13.0)	**0.03**
Yes		5 (27.8)	2 (11.1)	10 (55.6)	1 (5.6)	
**Means; linear regression, outcome** = **RCB Index**						
**Antibiotics use during IO**		***N***	**Mean (std)**	**Coefficient**		***P***
				**(95% CI)**^**a**^		
No		47	1.08 (1.41)	0 (Ref.)		
Yes		18	1.80 (1.43)	**0.86 (0.20–1.53)**		**0.01**
**Antibiotics use during IO**	**Outcome**	***N***	**%**	**OR (95% CI)**		***P***
				**(Outcome** = **PCR not achieved, unadjusted)**		
No	No PCR achieved	23	47.9	1 (Ref.)		0.08
	PCR achieved	25	52.1			
Yes	No PCR achieved	13	72.2	2.83 (0.87–9.17)		
	PCR achieved	5	27.8			

^a^Adjusted for age at screening for trial, HR status, and stage (II versus III).

^b^Fisher test.

## Discussion

We found that antibiotic exposure during IO was associated with a higher RCB among those with stage II/III BC. To our knowledge, this is the only study to describe an association of antibiotic usage with poorer clinical outcomes in patients with BC receiving neoadjuvant pembrolizumab in the curative-intent setting.

Our findings add to previous work which has suggested that the use of antibiotics during IO may be detrimental to treatment outcomes in advanced cancers^3–6^. Similar to a study that showed that antibiotic use at the same time as neoadjuvant pembrolizumab was associated with decreased rates of pCR and recurrence-free survival among patients with muscle-invasive muscle bladder cancer^7^, our study suggests that antibiotic use may also deter IO benefit in early-stage breast cancer.

Ours, together with previous studies suggest that the gut microbiome could act as a predictive biomarker for IO treatment response^3^. Modica et al. investigated the impact of the gut microbiome on trastuzumab antitumor efficacy in mice models of HER2-positive BC and found that the antitumor activity of trastuzumab decreased after fecal microbiota transplantation from antibiotic-treated donor mice^10^, suggesting a direct involvement of gut microbiome in the efficacy of trastuzumab as well. Future research should focus on elucidating the precise mechanism by which the gut microbiome modulates systemic and anti-tumor immunity.

Our study was limited by a small sample size and should be replicated in larger breast cancer studies. Higher RCB rates and lower pCR in the ATB+ group may be partly attributable to the cohort having medically sicker patients who required more antibiotics as opposed to the interplay between antibiotics and gut microbiome alone; however, we found no significant differences between the 2 groups with regard to exposure to pembrolizumab (number of doses or frequency of treatment delays). We did not analyze the results by the duration of antibiotics, their specific timing relative to IO, or their type, and did not assess the gut microbiome directly. More importantly, the temporal change in gut microbiome composition from baseline to post-antibiotic use in both groups is also lacking. Other unmeasured confounding factors that could potentially alter the gut microbiome composition are also not known. Future research should further investigate how combinations of other inter-connected factors like dietary intake, prebiotics, probiotics, prescription medications (e.g., proton pump inhibitors), and gastrointestinal infections (*Clostridium Difficile*), interact with antibiotics and affect the gut microbiome and responses to IO.

In summary, the use of antibiotics during neoadjuvant IO was associated with higher RCB. Larger studies are needed to assess the direct causal relationship between the gut microbiome and IO efficacy and shed light on the interplay between antibiotic exposure and IO to help optimize treatment outcomes.

## Methods

The I-SPY2 trial is an adaptive, randomized phase II trial (NCT#01042379) conducted in patients with high-risk, stage II/III BC. It has been previously described in detail^11,12^. We conducted a secondary analysis of participants in the pembrolizumab-4 arm: individuals with HER2-negative BC received pembrolizumab 200 mg every 3 weeks for four cycles concurrently with 80 mg/m^2^ paclitaxel weekly for 12 weeks, followed by four cycles of 60 mg/m^2^ doxorubicin plus 600 mg/m^2^ cyclophosphamide every 2 weeks. Of 69 participants, we excluded 2 individuals with stage I disease, and one with unknown antibiotics use. All participating sites received institutional review board approval, and patients provided written informed consent. The study complied with all relevant ethical regulations including the Declaration of Helsinki.

### Measures

The primary outcomes of our study were (a) RCB, with a higher RCB index indicating higher residual cancer burden and (b) pCR at the time of surgery.

The primary exposure of interest was antibiotic use during IO based on a review of the concurrent medication list in the study participants. Patients who received at least one dose of systemic antibiotics concurrently at the time of IO were included in the antibiotic exposure group (ATB+). All other participants were included in the control group (ATB-). In a supplemental analysis, we also explored whether the use of antibiotics outside of the ICI treatment window (ever use) affected primary outcomes.

### Statistical analysis

RCB index rates and pCR were compared between the ATB+ and ATB- groups using *t*-tests and Chi-squared tests, and linear and logistic regression models, respectively. Covariates for adjustment were defined a-priori: age at screening for the trial (years), stage of the tumor (II versus III), and hormone receptor (HR) status (HR+, HR–).

### Reporting summary

Further information on research design is available in the Nature Research Reporting Summary linked to this article.

### Supplementary information

SUPPLEMENTAL MATERIAL

Reporting Summary

## Data Availability

De-identified subject-level data are available to members of the research community by approval of the I-SPY Data Access and Publications Committee. Details of the application and review process are available at https://www.I-SPYtrials.org/collaborate/proposal-submissions.

## Code availability

Data analysis was performed using SAS 9.4. The codes generated and/or analyzed for the current study are available upon reasonable request and review.
