# Supplementary material for: Association of antibiotic exposure with residual cancer burden in HER2-negative early stage breast cancer
Source: NPJ Breast Cancer. 2024 Mar 26;10:24. doi: 10.1038/s41523-024-00630-w (PMC10966095; doi:10.1038/s41523-024-00630-w)
Supplement: Supplementary file 1 — SUPPLEMENTAL MATERIAL [file 41523_2024_630_MOESM1_ESM.pdf]

ISPY-2 Consortium members

Laura J Esserman, University of California San Francisco

Christina Yau, University of California San Francisco

Angela M. DeMichele, University of Pennsylvania

Nola M. Hylton, University of California San Francisco

Hope S. Rugo, University of California San Francisco

Jane Perlmutter, Gemini Group

W. Fraser Symmans, MD Anderson Cancer Center

Lamorna Brown-Swigart, University of California San Francisco

Rita Nanda, University of Chicago

Minetta C. Liu, Mayo Clinic Rochester

Rebecca Shatsky, University of California San Diego

Lajos Pusztai, Yale University

Anne Wallace, University of California San Diego

A. Jo Chien, University of California San Francisco

Erica Stringer-Reasor, University of Alabama at Birmingham

Erin Ellis, Swedish Cancer Center

Heather Han, Moffitt Cancer Center

Amy Clark, University of Pennsylvania

Kathy Albain, Loyola University Chicago Stritch School of Medicine

Judy C. Boughey, Mayo Clinic Rochester

Anthony Elias, University of Colorado

Claudine Isaacs, Georgetown University

Kathleen Kemmer, Oregon Health & Science University

Supplemental Table 1

Antibiotic class and name	Patients (N)
Aztreonam	1
Beta-lactams (amoxicillin, augmentin)	9
Cephalosporins (cefazolin, ceftriaxone and cephalexin)	16
Macrolides (azithromycin)	5
Nitrofurantoin	3
Fluoroquinolones (ciprofloxacin, levofloxacin)	16
Sulfonamides (bactrim)	7
Tetracyclines (doxycycline)	2
Vancomycin	2

Supplemental Table 2 Associations of antibiotics use (during or near-in-time with immunotherapy) with RCB index and pCR, unadjusted and adjusted, Pembrolizumab-4 arm of the I-SPY2 trial, N=66, 2015-2017

<table>
<tr><th colspan="6">Means; linear regression, outcome= RCB Index</th></tr>
<tr><th colspan="2">Antibiotics use during or near-in-time with IO</th><th>N</th><th>Mean (STD)</th><th>Coefficient (95% CI)*</th><th>P*</th></tr>
<tr><td colspan="2">No</td><td>26</td><td>1.00 (1.43)</td><td>0 (Ref.)</td><td></td></tr>
<tr><td colspan="2">Yes</td><td>39</td><td>1.47 (1.44)</td><td>0.45 (-0.18-1.08)</td><td>0.16</td></tr>
<tr><th colspan="6">Frequencies; logistic regression</th></tr>
<tr><th>Antibiotics use during or near-in-time with IO</th><th>Outcome</th><th>N</th><th>%</th><th>OR (95% CI) (Outcome= PCR not achieved, unadjusted)</th><th>P</th></tr>
<tr><td rowspan="2">No</td><td>pCR not achieved</td><td>12</td><td>44.4</td><td rowspan="2">1 (Ref.)</td><td rowspan="4">0.17</td></tr>
<tr><td>pCR achieved</td><td>15</td><td>55.6</td></tr>
<tr><td rowspan="2">Yes</td><td>pCR not achieved</td><td>24</td><td>61.5</td><td rowspan="2">2.00 (0.74-5.42)</td></tr>
<tr><td>pCR achieved</td><td>15</td><td>38.5</td></tr>
</table>

* Adjusted for age at screening for trial, hormone receptor status, and stage (I/II versus III)

Supplemental Table 3 Unadjusted associations of antibiotics use during immunotherapy with RCB index and pCR, by HR+ status, Pembrolizumab-4 arm of the I-SPY2 trial, N=65, 2015-2017

**Means; linear regression, outcome= RCB Index**					
**Hormone status**	**Antibiotics use during IO**	**N**	**Mean (STD)**	**Coefficient (95% CI)**	**P**
HR+	No	30	1.49 (1.51)	**0 (Ref.)**	**0.01**
	Yes	9	2.85 (0.98)	**1.36 (0.33-2.39)**	
HR-	No	17	0.35 (0.81)	0 (Ref.)	0.23
	Yes	9	0.76 (0.98)	0.41 (0.64-1.10)	

**Frequencies; logistic regression**						
**Hormone status**	**Antibiotics use during IO**	**Outcome**	**N**	**%**	**OR (95% CI) (Outcome= PCR not achieved, unadjusted)**	**P**
HR+	No	No PCR achieved	19	61.3		**0.04***
		PCR achieved	12	38.7		
	Yes	No PCR achieved	9	100	NA	
		PCR achieved	0	0		
HR-	No	No PCR achieved	4	23.5	1 (Ref.)	0.29
		PCR achieved	13	76.5		
	Yes	No PCR achieved	4	44.4	2.60 (0.46-14.6)	
		PCR achieved	5	55.6		

* Fisher exact test
