# Supplementary material for: Association of antibiotic exposure with residual cancer burden in HER2-negative early stage breast cancer
Source: NPJ Breast Cancer. 2024 Mar 26;10:24. doi: 10.1038/s41523-024-00630-w (PMC10966095; doi:10.1038/s41523-024-00630-w)
Supplement: Supplementary file 2 — Reporting Summary [file 41523_2024_630_MOESM2_ESM.pdf]

nature portfolio

Corresponding author(s): Amit Kulkarni

Last updated by author(s): Mar 5, 2024

Reporting Summary

Nature Portfolio wishes to improve the reproducibility of the work that we publish. This form provides structure for consistency and transparency in reporting. For further information on Nature Portfolio policies, see our Editorial Policies and the Editorial Policy Checklist.

Statistics

For all statistical analyses, confirm that the following items are present in the figure legend, table legend, main text, or Methods section.

n/a	Confirmed	
☒	☐	The exact sample size (*n*) for each experimental group/condition, given as a discrete number and unit of measurement
☒	☐	A statement on whether measurements were taken from distinct samples or whether the same sample was measured repeatedly
☐	☒	The statistical test(s) used AND whether they are one- or two-sided<br>*Only common tests should be described solely by name; describe more complex techniques in the Methods section.*
☐	☒	A description of all covariates tested
☒	☐	A description of any assumptions or corrections, such as tests of normality and adjustment for multiple comparisons
☐	☒	A full description of the statistical parameters including central tendency (e.g. means) or other basic estimates (e.g. regression coefficient) AND variation (e.g. standard deviation) or associated estimates of uncertainty (e.g. confidence intervals)
☐	☒	For null hypothesis testing, the test statistic (e.g. *F*, *t*, *r*) with confidence intervals, effect sizes, degrees of freedom and *P* value noted<br>*Give P values as exact values whenever suitable.*
☒	☐	For Bayesian analysis, information on the choice of priors and Markov chain Monte Carlo settings
☒	☐	For hierarchical and complex designs, identification of the appropriate level for tests and full reporting of outcomes
☒	☐	Estimates of effect sizes (e.g. Cohen's *d*, Pearson's *r*), indicating how they were calculated

Our web collection on statistics for biologists contains articles on many of the points above.

Software and code

Policy information about availability of computer code

Data collection	The study case report forms (CRF) are the primary data collection instrument for the study. The CRFs for this study was completed electronically using a RedCap database system accessible to all study sites.
Data analysis	SAS 9.4 software was used for data analysis.

For manuscripts utilizing custom algorithms or software that are central to the research but not yet described in published literature, software must be made available to editors and reviewers. We strongly encourage code deposition in a community repository (e.g. GitHub). See the Nature Portfolio guidelines for submitting code & software for further information.

Data

Policy information about availability of data

All manuscripts must include a data availability statement. This statement should provide the following information, where applicable:

- Accession codes, unique identifiers, or web links for publicly available datasets
- A description of any restrictions on data availability
- For clinical datasets or third party data, please ensure that the statement adheres to our policy

De-identified subject level data are available to members of the research community by approval of the I-SPY Data Access and Publications Committee. Details of the application and review process are available at https://www.I-SPYtrials.org/collaborate/proposal-submissions.

Research involving human participants, their data, or biological material

Policy information about studies with human participants or human data. See also policy information about sex, gender (identity/presentation), and sexual orientation and race, ethnicity and racism.

Reporting on sex and gender	The reporting on sex and gender is not directly relevant to the study since only women with breast cancer diagnosis were included.
Reporting on race, ethnicity, or other socially relevant groupings	In the manuscript we have not used any socially relevant categorization variables. The self-reported ethnicity data in the same trial participants is available in a previous manuscript that is publicly accessible Nanda R et al JAMA Oncol. 2020 May; 6(5): 1–9.
Population characteristics	Patients eligible for I-SPY2 are women aged 18 years or older, with stage II or III breast cancer and primary tumors larger than 2.5 cm by clinical examination or larger than 2.0 cm by imaging, and Eastern Cooperative Oncology Group performance status of 0 or 1. Patients with MammaPrint low-risk HR-positive, ERBB2-negative disease are excluded from I-SPY2 because their lower risk of recurrence does not justify escalation of therapy. Only ERBB2-negative patients were eligible for randomization to the pembrolizumab arm.
Recruitment	Participant eligibility was systematically assessed at each of the participating I-SPY 2 study sites. A screening log was kept in TRANSCEND documenting the review of potentially eligible participants as well as reasons for non-enrollment. Sites will provide detailed information to all relevant treating physicians on the conduct of the trial to optimize physician participation. Monthly conference calls will review recruitment at each site so that sites not meeting recruitment goals can be identified early and interventions to improve recruitment can be instituted.<br>The I-SPY 2 TRIAL is listed on the NIH website Clinicaltrials.gov to enable referring physicians to identify local sites for participant referral (NCT01042379). Participants will also be able to find the I SPY-2 TRIAL sites through breastcancertrials.org, a clinical trial matching web site. Women diagnosed with breast cancer can go to this national-service web site and enter their information to find clinical trials appropriate for them. Once they find a trial, they can contact a research site and send their information through TRIAL CONNECT, which includes their contact information and their eligibility screened against the trial eligibility. In addition, the study will work with advocate groups across the country to improve awareness.<br>A large, organized cadre of experienced participant advocates participated within community participant support services locations to help educate participants about the trial and assist in the recruitment and retention process. These advocates will be experts on the trial design and conduct and will assist potentially eligible participants in understanding the informed consent process as well as assisting those enrolled in navigating the various steps within the trial assessment and treatment process.
Ethics oversight	The trial was designed by the I-SPY2 study investigators. Merck provided study drug but played no role in the study design, collection/analysis of data or manuscript preparation. All participating sites received institutional review board approval, and patients provided written informed consent. The I-SPY2 DSMB meets monthly to review patient safety and study progress.

Note that full information on the approval of the study protocol must also be provided in the manuscript.

Field-specific reporting

Please select the one below that is the best fit for your research. If you are not sure, read the appropriate sections before making your selection.

☒ Life sciences ☐ Behavioural & social sciences ☐ Ecological, evolutionary & environmental sciences

For a reference copy of the document with all sections, see nature.com/documents/nr-reporting-summary-flat.pdf

Life sciences study design

All studies must disclose on these points even when the disclosure is negative.

Sample size	Sample size calculation for this analysis is not applicable.
Data exclusions	ISPY-2 enrolled patients with high-risk early-stage breast cancer (Stage II and III only) and primary tumors larger than 2.5 cm by clinical examination or larger than 2.0 cm by imaging. We excluded 2 patients who had clinical (T) size of 2 cm or less based on the longest diameter of lesion by clinical palpation at baseline but met the criteria for inclusion based on longest diameter by MRI assessment at baseline. This criteria was established before analysis was undertaken.
Replication	The analysis was reproducible when done independently.
Randomization	Although the data is from a randomized clinical trial, the analysis described here in is not applicable directly as it is data from only one of the arms.
Blinding	Not applicable.

Reporting for specific materials, systems and methods

We require information from authors about some types of materials, experimental systems and methods used in many studies. Here, indicate whether each material, system or method listed is relevant to your study. If you are not sure if a list item applies to your research, read the appropriate section before selecting a response.

Materials & experimental systems

n/a	Involved in the study
☒	☐ Antibodies
☒	☐ Eukaryotic cell lines
☒	☐ Palaeontology and archaeology
☒	☐ Animals and other organisms
☐	☒ Clinical data
☒	☐ Dual use research of concern
☒	☐ Plants

Methods

n/a	Involved in the study
☒	☐ ChIP-seq
☒	☐ Flow cytometry
☒	☐ MRI-based neuroimaging

Clinical data

Policy information about clinical studies

All manuscripts should comply with the ICMJE guidelines for publication of clinical research and a completed CONSORT checklist must be included with all submissions.

Clinical trial registration	NCT01042379
Study protocol	Trail protocol is publicly available and can be accessed from a prior publication of the study Nanda R et al JAMA Oncol. 2020 May; 6(5): 1–9.
Data collection	The patients were enrolled in the institutions participating in the ISPY-2 trial. Patients were enrolled on the study between November 26, 2015, and November 5, 2016.
Outcomes	The primary outcomes of our study were a) Residual Cancer Burden (RCB), with a higher RCB index indicating higher residual cancer burden and b) Pathologic complete response (pCR). Both were determined by the pathologist at the time of surgery. RCB and PCR were both measured as previously described by Symmans et al J Clin Oncol. 2007;25(28):4414-4422.

Plants

Seed stocks	NA
Novel plant genotypes	NA
Authentication	NA
